# Location, age, and race matter: a path model of emotional distress in the U.S. during COVID-19

**DOI:** 10.1186/s12889-023-15640-9

**Published:** 2023-04-25

**Authors:** Viktor Clark, Hannah Ming, Sunny Jung Kim

**Affiliations:** 1 Department of Health Behavior and Policy, School of Medicine, Virginia Commonwealth University, 830 East Main Street, 4th Floor, Richmond, VA 23298-0430 USA; 2 Massey Cancer Center, Virginia Commonwealth University, Richmond, VA 23298 USA

**Keywords:** COVID-19, Mental health, Racial and ethnic minority populations, Rural health, Social determinants of health

## Abstract

**Background:**

We aim to identify factors that explain emotional distress among underserved populations during the COVID-19 pandemic.

**Methods:**

Starting in August 2020, we conducted an online epidemiological survey among 947 U.S. adults. The survey asked a wide array of constructs, including demographics, past-month substance use, and psychological distress. We developed a path model to understand how financial strain, age, and substance use are associated with emotional distress among People of Color (POC) and those living in rural areas.

**Results:**

22.6% (*n* = 214) of participants were POC; 114 (12%) resided in rural areas; 17.2% (*n* = 163) made between $50,000 and $74,999 annually; and the emotional distress average was 1.41 (SD = 0.78). POC, especially those younger, experienced higher rates of emotional distress (*p* < .05). People living in rural contexts reported lower rates of emotional distress through low alcohol intoxication and less financial strain (*p* < .05).

**Conclusions:**

We found mediating factors related to emotional distress among vulnerable populations during the COVID-19 pandemic. Younger POC experienced higher rates of emotional distress. People in rural communities had less emotional distress when they had fewer days spent intoxicated by alcohol, which was associated with lower financial strain. We conclude with a discussion of important unmet needs and future research directions.

## Introduction

### COVID-19

On December 31, 2019, the first patient presented with an unknown cause of pneumonia in Wuhan, China [1]. It was soon discovered that this unknown cause was going to be the first case of what is known globally as severe acute respiratory syndrome-coronavirus-2 (SARS-COV-2), which would lead to the pandemic corona virus and disease 2019 (COVID-19) [1]. The World Health Organization officially declared COVID-19 a global pandemic on March 11, 2020 [1]. As of September 2020, the cases of COVID-19 in the U.S. reached 2 million, and over 650,000 deaths were reported during this first wave of the pandemic [2].

### Factors contributing to emotional health disparities

#### Effects of being a person of color

Prior to the COVID-19 pandemic, there were minimal disparities in prevalence of mental illness based on race/ethnicity according to the U.S. Census Data, 2017 [3]. Despite similar prevalence rates, help-seeking behaviors for mental illness are found to be lower in communities among People of Color in the U.S [4]. However, since the pandemic, research has indicated that both Black and Hispanic/Latinos have carried a disproportionate rate of psychological distress compared to White non-Hispanic Americans [5], with one study reporting that Hispanic/Latinos face double the rates of depression, four times the rates of suicidal thoughts/ideation, and more than two times the rates of initiation or increase in substance use, compared to White and Black non-Hispanics/Latinos [6]. While race and ethnicity can explain some of the disparities in emotional distress that have resulted from the COVID-19 pandemic, other determinants should be considered to gain a holistic picture of whose mental health has been disproportionately affected by the pandemic. There are also additional disparities racial and ethnic minorities experience such as financial burden which have been exasperated by the COVID-19 pandemic. According to a national survey administered during July and August 2020, 72% of Latino and 60% of Black Americans experienced severe financial hardship during the pandemic [7]. These financial hardships included the inability to pay mortgage or rent, inability to pay for food or groceries, and a decline in overall household income [7]. Financial strain is one facet of the disparities that both People of Color and people living in rural areas in America have experienced.

#### Effects of living in Rural America

There are currently 60 million people in the U.S. living in rural America [8]. Among people living in rural areas, poor emotional health has been found to be a determinant of higher rates of suicidality and substance use, specifically before the COVID-19 pandemic [9–11]. During the pandemic much research has centered around the emotional health status of those living in urban context, and the evidence for rural mental health is limited. One study found that 43.68% of survey respondents living in rural areas experienced negative effects on their mental health due to COVID-19 [7]. Additionally, people living in rural areas of America have carried significant financial burden due to the COVID-19 pandemic [12]. Specifically, a survey focused on Western rural areas in the U.S. reported rates of only 21% being full-time employees prior to the pandemic and approximately 50% of those full-time employees reported temporary unemployment since March 2020 [4]. This survey also identified that, of the 41.07% of participants who were working part-time prior to the pandemic, 72.77% faced temporary unemployment [4]. The psychological toll that has resulted from the pandemic and financial constraints has been devastating to rural America. Therefore, in the present study, we examine the extent to which living in rural context along with age, financial hardship, and alcohol use contribute to emotional distress during the pandemic.

#### Effect of age

There are currently over 50 million adults aged 65 and older living in the U.S. The demographic distribution of this population differs significantly based on race; Black/African Americans aged 65 and older accounted for 11.36% of Black/African American U.S. populations; and Hispanics aged 65 and older accounted for 7.34% of Hispanic U.S. populations; compared to White Americans aged 65 and older being 17.36% of the White U.S. populations [13]. Additionally, among a national sample from 2018, 17.5% of people living within rural contexts were 65 years or older [14]. As of 2021, approximately 60% of all COVID-19 deaths were among adults ages 65 and older [15]. While the older American population has experienced much of the death toll due to COVID-19, younger adults have faced the highest drops in unemployment rates: 15–24-year-olds accounted for 50.71% of the working adult force in April 2019 and dropped to 35.99% in April 2020; and 25–54-year-olds made up nearly 80% of the working force in April 2019 and dropped to less than 70% in April 2020 [16]. These death toll and unemployment rates indicate negative consequences of the pandemic affecting various age groups in different ways.

During the COVID-19 pandemic, specifically due to lockdown and closures, rates of emotional distress among young adults (18–34-year-olds) were found to significantly increase [17]. While age itself has been found to be a significant predictor of emotional distress, determining the role of age with other social determinant factors, such as rurality, race/ethnicity and financial strain, will further extend our understanding of detrimental effects of COVID-19.

#### Effect of financial strain

In addition to living in rural areas and being a person of color, the extent of the financial strain experienced has been found to be related to emotional distress [18]. At the end of 2020, there was a total of 10.8 million people in the U.S. that were unemployed [19]. Unemployment has placed burden on people’s ability to maintain various basic necessities such as housing, food, and health insurance [5]. Among those who have experienced financial strain, depressive symptoms continued to rise across the duration of the pandemic, especially for those with a continuance of financial strain since the onset of COVID-19 in the U.S [20].

#### Effect of alcohol use

Negative affect, described as feeling distressed or sad, has been determined as a significant predictor of craving substances, particularly alcohol [21]. During the COVID-19 pandemic there has been an alarming increase in reported substance use and overdose deaths especially among vulnerable populations. Between People of Color and White adults, rates of substance use in the US have been comparable [22]. However, what triggers the use of substances such as alcohol among People of Color versus White adults differs by distinct mechanisms, such as discrimination and stigma being strong predictors for substance use among People of Color [23]. Substance use among people living within a rural context in America has been well established with higher rates in rural settings compared to urban settings [11]. Age has also been found to be a significant predictor of alcohol use; specifically, young adults ages 21–23 have been found to engage in heavy or binge drinking as a means to cope with suicidal ideation [24]. Finally, financial strain, specifically that of high financial strain due to job loss was found to significantly predict alcohol use among adults in America [25].

While each of these determinants (race/ethnicity, rurality, age, financial strain, and substance use) independently have been found to impact emotional health, examining the intersectionality and mediating effects these determinants have on mental health is essential when developing policies and interventions on pandemic responses. To date there has yet to be a single model that evaluates the directions and magnitudes of these factors (being a Person of Color, living in rural America, age, enduring financial strain, and alcohol use) toward emotional distress.

#### The current study

**Fig. 1:**
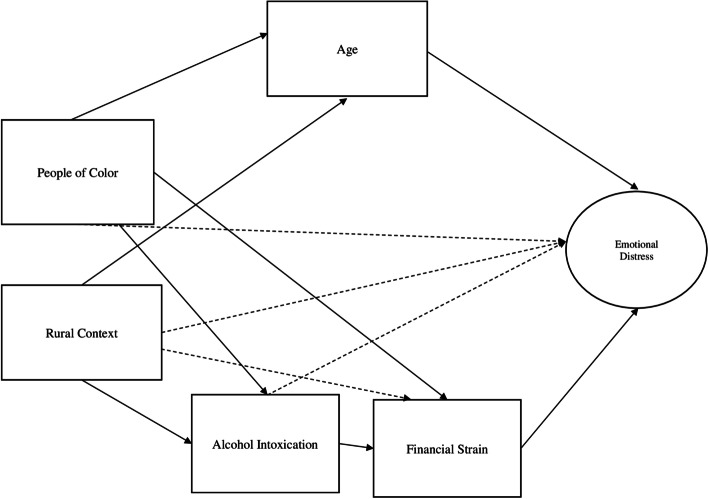
Path model

The current study will employ an intersectional lens to explore the pathways by which and to what extent race/ethnicity, rurality, age, substance use, and financial strain influence experiences of emotional distress within the context of COVID-19. We developed a structural equation model (see Fig. 1) to illustrate the pathways that are significantly related to emotional distress. We propose that:

*Hypothesis 1*: People of Color are more likely to experience emotional distress because of indirect effects of age, financial strain, and alcohol intoxication. This is demonstrated by literature indicating that both Black and Hispanic/Latinos have carried a disproportionate rate of psychological distress compared to White non-Hispanic Americans since the onset of the COVID-19 pandemic [5], and Hispanic/Latinos have been found to have more than two times the rates of initiation or increase in substance use since the COVID-19 pandemic, compared to White and Black non-Hispanics/Latinos [6]. This study will further explore if age and financial strain are associated with the experiences of emotional distress particularly to People of Color.

*Hypothesis 2*: People living in rural contexts will experience higher levels of emotional distress due to the indirect effects of age, financial strain, and alcohol intoxication. People living in rural areas have been found to experience negative effects of mental health compared to those living in urban areas since the onset of the COVID-19 pandemic [7]. This study will explore further if age, financial strain, and alcohol intoxication relate to levels of emotional distress.

## Methods

### Ethics statement

This study was approved by the institutional review board at Virginia Commonwealth University (IRB HM20019183).

### Participant consent

Individuals who wanted to participate in the survey were asked to read the information of the study, click the “Yes, I agree” button and the “Next” button at the bottom of the information sheet to complete the implied consent process. This informed consent process was approved by the ethics committee at Virginia Commonwealth University. Participants had the option to respond to all questions, or refuse to answer any question, and still receive their full compensation. Finally, participants had the PI's contact information for any questions or concerns that arose prior to or after their participation in the survey.

### Participants

Between August and December 2020, we distributed an epidemiologic survey through multiple online platforms including Amazon Mechanical Turk and Qualtrics Panel [26]. Informed consent was collected prior to participants engaging in the survey. The eligibility criteria included participants living within the U.S., being 18 years of age or older, and having proficiency in English. From this survey, we recruited 947 participants, 865 participants (91%) completed all items utilized within our analyses.

### Measures

The epidemiologic survey asked a wide array of constructs, including age, sex (male or female), race comprised of 15 categories and ethnicity comprised of 5 categories, which were then dummy coded into a dichotomous categorical variable (People of Color = 1, non-Hispanic White = 0). If a participant indicated their race was non-White, regardless of their ethnicity, participants were categorized as a Person of Color [27]. We also measured other social determinant factors, such as zip code which was converted into a RUCA code (Version. 2019) and dichotomized into either an urban or rural living context (rural-living = 1, urban-living = 0) [28], income level consisting of 9 categories, education level consisting of 7 categories, and occupational status consisting of 7 categories. The Epidemic Pandemic Impacts Inventory (EPII) 4-item economic subscale indicating financial strain was used with responses of either ‘yes’ or ‘no’ endorsing or refuting each item (e.g., “unable to get enough food or healthy food”); higher scores indicate more financial strain [29]. Borrowed from the Addiction Severity Index scale, alcohol intoxication was measured by an open-ended item, “how many times over the past 30 days did you become intoxicated using alcohol? [30]”.

**Fig. 2:**
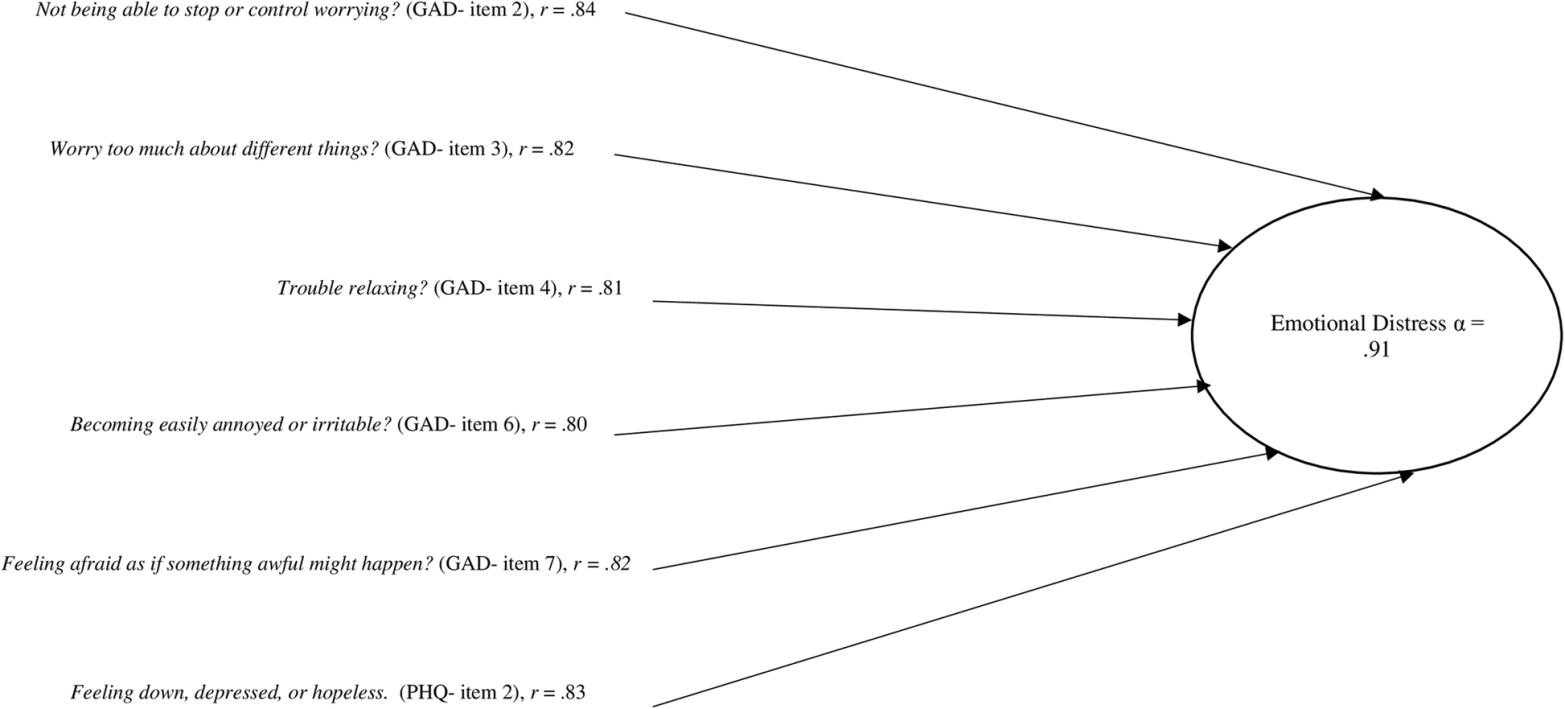
Confirmatory factor anlaysis: emotional distress

For the final endogenous variable (i.e., emotional distress), we assessed level of anxiety using the General Anxiety Disorder (GAD)-7 (e.g., “Feeling nervous”) [31], as well as depression by using the Patient Health Questionnaire (PHQ)-2 scale (e.g., “Little interest or pleasure in doing things”) [32]. For the GAD-7 items, respondents indicated the occurrence of items on a 4-point Likert scale with values ranging from ‘0- Not at all’ to ‘3-Nearly every day’ (α = 0.93); higher scores indicate higher levels of anxiety. For the PHQ depressions items, respondents indicated the occurrence of each item with a 4-point Likert scale, ranging from ‘0-Not at all’ to ‘3- Nearly every day’ (α = 0.81); higher scores indicate higher levels of depression. We developed a single scale through a Confirmatory Factor Analysis (see Fig. 2) to measure emotional distress by combining high factor loading items of the GAD-7 and PHQ-2 (individual item factor loading coefficients > 0.80) for a composite α = 0.91, with higher scores indicating greater emotional distress. Previous literature has indicated that both anxiety and depression are emotionally distressing experiences therefore, a concise variable combining these experiences was deemed appropriate [33].

#### Data analysis

Descriptive chi-square tests were run comparing rates of People of Color and rurality on all categorical variables, including sex, income, education, and occupational status with a significance level of 0.05. Additionally, descriptive mean comparisons for all continuous variables (age, anxiety, depression, financial strain, emotional distress, and alcohol intoxication) between groups (People of Color and rurality) were conducted by a Student’s t-test. To capture all types of positive or negative relationships between variables used in the model, we conducted Spearman correlation using a heatmap. We used the function “corrplot” from the package corrplot() to visualize a Spearman correlation matrix [34]. To test our hypotheses, we developed a path model to explore the relationships between age, and alcohol intoxication by People of Color and rurality in predicting the variance of emotional distress (Fig. 1). We first associated each of the variables with one another and examined our model fit indices. We then modified the paths based on significance levels and fit indices to develop the most parsimonious and best fitting model. AMOS 23.0 and IBM SPSS 27.0 were used to conduct our analyses.

## Results

### Differences between sub-groups

The average age of the sample was 39.11 *(SD* = 12.85), and majority of the sample were identified as female (*n* = 505, 53.3%); 24.6% of the sample were coded as being a Person of Color (*n* = 233); 12.04% of the sample indicated they lived in a rural context (*n* = 114); 50.3% of our sample made under $50,000 a year (*n* = 440); 37.1% of the sample had graduated from college (*n* = 325); and 27.7% of participants were unemployed at the time of this survey (*n* = 76). From chi-square tests, we found that people living in a rural context were more likely to be women (*X*^2^ = 6.55, *p* = 0.01). People of Color were found to make significantly less money than White participants (*X*^2^ = 24.97, *p* = 0.002). People living in rural context were found to make significantly less money than people living in urban context (*X*^2^ = 19.22, *p* = 0.01). People living in a rural context had significantly lower levels of education than people living in an urban context (*X*^2^ = 19.72, *p* = 0.003). Finally, People of Color were significantly less likely to be employed compared to White participants (*X*^*2*^ = 19.95, *p* = 0.003). Similarly, people living in rural context were significantly less likely to be employed compared to people living in an urban context, *X*^*2*^ = 25.89, *p* < 0.001 (see Table 1 for full sample characteristics).

**Table 1:** Sample Characteristics

	*µ (SD) or n (%)* ***People of Color (a)***	*µ (SD) or n (%)* ***White*** ***(ref,)***	*µ (SD) or n (%)* ***Rural Context (b)***	*µ (SD) or n (%)* ***Urban Context (ref,)***	*µ (SD) or n (%)* ***Overall***
**Age (a**^**+**^**) (b)**	36.02 (12.09)^+^	40.12 (12.94)	38.58(12.16)	39.27 (12.94)	39.11 (12.85)
Sex (a) (b^*^)					
Male	102(43.8%)	340 (47.6%)	48 (42.1%)	371 (45.1%)	442 (46.7%)
Female	131 (56.2%)	374 (52.4%)	66 (57.9%)	451 (54.9%)	505 (53.3%)
Income (a^*^) (b^*^)					
$0-$9,999	28 (12.8%)	45 (6.8%)	11 (10.3%)	61 (8.0%)	73 (7.7%)
$10,000-$14,999	16 (7.3%)	36 (5.5%)	9 (8.4%)	43 (5.7%)	52 (5.5%)
$15,000-$19,999	12 (5.5%)	35 (5.5%)	10 (9.3%)	36 (4.7%)	47 (5.0%)
$20,000-$34,999	38 (17.4%)	93 (14.2%)	19 (17.8%)	111 (14.6%)	131 (13.8%)
$35,000-$49,999	42 (19.3%)	95 (14.5%)	19 (17.8%)	117 (15.4%)	137 (14.5%)
$50,000-$74,999	40 (18.3%)	123 (18.7%)	18 (16.8%)	141 (18.6%)	163 (17.2%)
$75,000-$99,999	18 (8.3%)	99 (15.1%)	16 (15.0%)	99 (13.1%)	117 (12.4%)
$100,000-$199,999	17 (7.8%)	101 (15.4%)	5 (4.6%)	113 (14.9%)	118 (12.5%)
$200,000 or more	7 (3.2%)	30 (4.3%)	0 (0.0%)	37 (4.9%)	37 (3.9%)
Education (a) (b^*^)					
< 8 Years	10 (4.6%)	15 (2.3%)	3 (2.8%)	21 (2.8%)	25 (2.6%)
8–11 Years	14 (6.4%)	31 (4.7%)	6 (5.6%)	38 (5.0%)	45 (4.8%)
12 Years or High School	35 (16.1%)	108 (16.4%)	24 (22.4%)	118 (15.6%)	143 (15.1%)
Post HS training outside College	4 (1.8%)	16 (2.4%)	4 (3.7%)	15 (2.0%)	20 (2.1%)
Some College	34 (15.6%)	115 (17.5%)	26 (24.3%)	122 (16.1%)	149 (15.7%)
College Graduate	91 (41.7%)	234 (35.6%)	38 (35.5%)	283 (37.3%)	325 (34.3%)
Postgraduate	30 (13.8%)	138 (21.1%)	6 (5.6%)	161 (21.2%)	168 (17.7%)
Occupational Status (a^*^) (b^**+**^**)**					
Employed	150 (68.8%)	483 (73.5%)	60 (56.1%)	566 (74.7%)	633 (66.8%)
Unemployed	27 (12.4%)	49 (7.5%)	19 (17.8%)	56 (7.4%)	76 (8.0%)
Homemaker	10 (4.6%)	45 (6.8%)	6 (5.6%)	49 (6.5%)	55 (5.8%)
Student	16 (7.3%)	15 (2.3%)	5 (4.7%)	25 (3.3%)	31 (3.3%)
Retired	10 (4.6%)	41 (6.2%)	9 (8.4%)	41 (5.4%)	51 (5.4%)
Disabled	4 (1.8%)	18 (2.7%)	7 (6.5%)	15 (2.0%)	22 (2.3%)
Other	1 (0.5%)	6 (1.0%)	1 (0.9%)	6 (1.0%)	7 (0.7%)
**Alcohol Intoxication (Days Used) (a) (b)**	3.54 (6.60)	3.15 (6.04)	1.43 (5.48)	3.48 (6.23)	3.24 (6.16)
Anxiety (GAD-7) (a^*^) (b)	1.40 (0.86)	1.24 (0.89)	1.29 (0.86)	1.27 (0.89)	1.28 (0.88)
Depression (PHQ-2) (a^*^) (b)	1.38 (0.95)	1.19 (0.96)	1.16 (0.97)	1.24 (0.96)	1.24 (0.96)
Financial Strain (EPII) (a) (b^*^)	0.33 (0.39)	0.29 (0.36)	0.23 (0.35)	0.31 (0.37)	0.30 (0.37)
Emotional Distress (a^*^) (b)	1.52 (0.73)	1.38 (0.79)	1.40 (0.77)	1.41 (0.78)	1.41 (0.77)
**Total**	233 (24.60%)	714 (75.40%)	114 (12.04%)	833 (87.96%)	947 (100%)

*p < .05 +*p* < .001

X2-tests were conducted for all categorical variables

Student’s t-tests were conducted for all continuous variables

We then proceeded to compare the mean scores through our Student’s t-tests. It was determined that average anxiety levels were found to be significantly higher among People of Color compared to White participants, *t* = 2.33, *p* = 0.02, 95% CI (0.03, 0.30). Average levels of depression were found to be significantly higher in People of Color compared to White participants, *t* = 2.53, *p* = 0.01, 95% CI (0.04, 0.34). Average levels of emotional distress were found to be significantly higher among People of Color compared to White participants, *t* = 2.27, *p* = 0.02, 95% CI (0.02, 0.25). Finally, financial strain was found to be significantly lower among people living within a rural context compared to people living within an urban context, *t* = -1.95, *p* = 0.05, 95% CI (-0.15, 0.00). We summarized all descriptive statistics in Table 1.

#### Spearman correlation

**Fig. 3:**
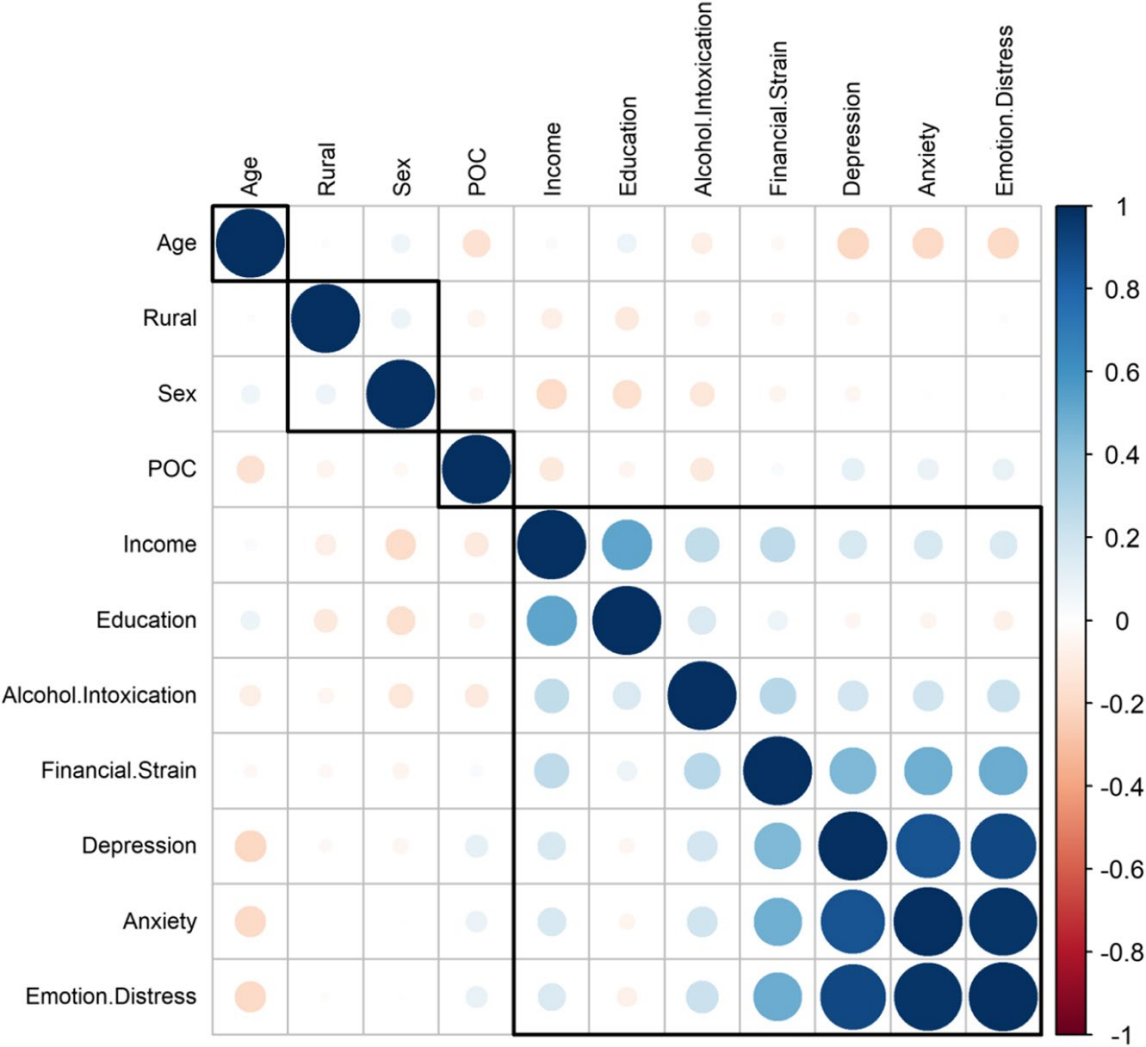
A heatmap with the possible Spearman correlation of the selected variables. Sex (1=male, 2=female), Rural (rural =1, urban=0), POC (1=POC, 0=non Hispanic White). For the variables, income and education, greater values indicate greater income and higher education

The correlation heatmap (Fig. 3) displays a gradient from red to blue for lower correlations of variables to higher correlations. Variables with similar patterns of Spearman correlation coefficients were clustered closer together to effectively visualize the distribution of correlations. Financial strain was positively correlated with anxiety, depression and emotional distress, whereas age (being older) was negatively associated with these variables.

### Path model outcomes

**Fig. 4:**
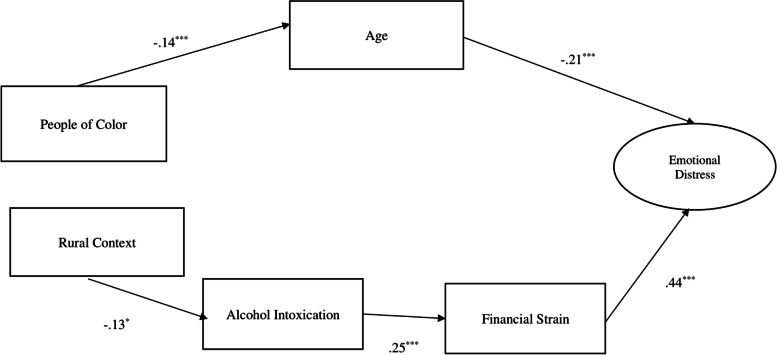
Final path model Coefficients are standardized Alcohol Intoxication, #of Days over 30 days Intoxicated Alcohol *p<.05***p<.05.001 Fit Indices: NFI: .959 RFI: .914 IFI: 0.995 TLI: .989 CFI: 0.995 RMSEA: .012

Our initial model was developed by creating pathways between each variable exploring every relationship possible among the variables (as proposed in Fig. 1). The Chi-square of our first iteration of the model was non-significant, indicating a moderate model fit, *X*^2^_2_ = 1.29, *p* = 0.52. There were no significant relationships for People of Color and days intoxicated, levels of financial strain, or levels of emotional distress, *p*= n.s. For people living within a rural context, there were no significant relationships between being a Person of Color, financial strain, and emotional distress. Finally, age did not have significant relationships with days intoxicated or level of financial strain. Based on previous literature and path coefficients of the initial model, we removed non-significant paths in order to develop the most parsimonious model [35]. This modified path model had a good fit (NFI = 0.959, CFI = 995, *X*^2^_10_ = 11.37, *p* = 0.33, RMSEA = 0.012, Fig. 4).

People of Color were significantly younger than White participants, and younger adults were more likely to report greater emotional distress (*p* < 0.001). This pathway indicated that the relationship with emotional distress and being a Person of Color was significantly mediated by age, *B* = 0.03, partially supporting Hypothesis 1.

Rurality and days intoxicated were found to be related, indicating that those living within a rural context on average spent 2.5 days less in the past 30 being intoxicated compared to people living in non-rural areas, *p* = *0.0*2. Days intoxicated were found to be significantly related to financial strain, implying that as days intoxicated increased financial strain also increased (*p* < 0.001). Financial strain was significantly related to emotional distress, demonstrating that as financial strain increased emotional distress increased (*p* < 0.001). That is, people living in rural areas had lower emotional distress compared to people in non-rural areas, which was mediated by the lower number of days intoxicated which influenced lower financial strain, *B* = -0.01. This pathway is contradictory to the overall direction of our Hypothesis 2, which posited that people living in a rural context will experience higher levels of emotional distress due to the indirect effects of age, financial strain, and alcohol intoxication (See Fig. 4 for final path model and fit indices).

## Discussion

The overall aim of this paper was to understand the mechanism constructs that contribute to emotional distress. We especially focused on vulnerable communities such as People of Color and people living in rural areas, as well as the way diverse age groups have been impacted by the COVID-19 pandemic. We first ran a correlation heatmap to visualize correlation patterns between variables. The heatmap confirmed that being younger, being a Person of Color, and having financial strain were positively associated with emotional distress. In testing our hypotheses, we found two significant pathways with distinct mechanism constructs: first, People of Color had greater emotional distress and this relationship was mediated by age (being younger); second, people living in rural contexts had better emotional health compared to people in non-rural contexts, and this association was explained by the lower number of days intoxicated, which was associated with lower financial strain. We found several benefits in utilizing path analysis for our study. First, utilizing path analysis allowed us to demonstrate the comparative levels of emotional distress among two vulnerable populations—POC and people living in rural areas-simultaneously [35]. Second, our study was primarily interested in determining the mechanisms (age, alcohol consumption, financial strain) that impact the level of emotional distress POC and people living in rural areas experience. Therefore, through path analysis, we were able to examine multiple variables at one time that directly or indirectly impacted levels of emotional distress, which is not feasible using Analysis of Variance tests [35].Lastly, by using the path modeling framework, we were able to retain paths that were significant while reducing those non-significant paths to create a parsimonious model that explained significant levels of variance in emotional distress for the unique lived experiences of POC and people living in rural areas within our study [35]. Ultimately these benefits made path analysis a clear superior method over a regression model to test our hypotheses.

Determining what may be impacting emotional distress during COVID-19 has been a focus for many studies since the onset of the pandemic [36–38]. Our findings that age, specifically being younger, mediates the relationship between being a POC and emotional distress are similar to findings from other studies where being a younger adult led to higher levels of psychological stress related to COVID-19 [36]. These differences may be explained by disparities in how young people are coping with stress that is related to COVID-19 [36]. In discussing our second path (people living in rural areas, alcohol consumption, financial strain, and emotional distress), multiple studies have reported an increase in alcohol consumption during the COVID-19 pandemic [37, 38]. However, for some, alcohol consumption has reportedly decreased due to a lack of available finances, inaccessibility to alcohol, and less leisure time [36]. Our study is consistent with these earlier findings as, among individuals living in rural areas, less alcohol use led to less financial strain and ultimately less emotional distress.

Our study also found some nuanced results. Based on previous literature we predicted that People of Color would face significant levels of financial strain; this was not supported [7]. Similarly, despite literature indicating that People of Color may use substances to cope with emotional distress, this present study found no such relationship [24]. One of the most surprising findings was that people living in a rural context were found to have lower levels of emotional distress compared to people living in urban areas (reference group), while multiple sources have indicated finding the opposite [7, 9–11]. To help explain these mixed findings, a new assets-based model, the Minority Strengths Model, was developed with psychosocial constructs such as resilience, social supports, and self-esteem and has been found to significantly predict mental health [39].

### Limitations

While this study utilized some strong methodological and analytical techniques such as the utilization of constructs with high internal validity, including our outcome variable, and the utilization of an exploratory path model analysis that allowed for multiple paths and comparative analyses, our study still had some limitations imposed by the study design. Our study was cross-sectional in nature and based on a survey. By nature, a study with multiple time point assessments, and using an experimental design would help us understand a trend over time and lead to higher levels of causal inferences. A future study may explore the impacts of COVID-19 through a longitudinal design or a case–control study design. Second, our sample was recruited using online panels and crowdsourcing. This method for recruitment has been found to lead to strong samples compared to traditional convenience sampling [26]; however, if participants do not have access to the internet, their opportunity for inclusion may be eliminated. Finally, as far as our subsample sizes and analysis capability, our sample entailed 24.6% People of Color which is consistent with the U.S. Census Bureau: [40] however, our sample of people living in a rural context slightly underrepresented (12.04%) the nearly 20% of the U.S. population.

### Recommendations

We recommend a few directions for future studies. First, future research may replicate this survey now since vaccinations have been introduced and people may be returning to work, which may ultimately impact their levels of financial strain and overall emotional distress. Second, conducting a longitudinal study to examine at-risk communities’ burden from the pandemic would be recommended to determine long-term results of the pandemic. Third, developing targeted interventions for younger communities, communities of color, or rural communities to reduce emotional distress and substance use particularly during a pandemic may be an important strategy to promote health. Additionally, while this study aimed at identifying communities at risk of facing adversity due to the COVID-19 pandemic and the outcome of those adversities, an expansion of this understanding would be to identify what factors may have protected at-risk communities from developing negative outcomes such as emotional distress [38]. Similarly, developing targeted interventions for younger communities, communities of color, or rural communities to reduce emotional distress and substance use particularly during a pandemic may be an effective strategy to promote public health. Fourth, machine learning is a growing field of research methodology particularly surrounding forecasting models for various occurrences including spread of the COVID-19 pandemic, natural disasters, and emotional distress [41–45]. Future research may develop and apply a machine learning algorithm to best identify high-risk populations for emotional distress during a pandemic or other troubling life experience and tailor interventions to reduce this distress. Finally, future research may perform epidemiological research like ours outside of the United States to understand additional intersections and nuances concerning the impact of global pandemics on emotional distress.

## Conclusions

Our study demonstrates evidence for disparities among People of Color, specifically those who are younger facing higher rates of emotional distress. We also found that people living in rural contexts who spent less days intoxicated by alcohol reported less financial strain, which ultimately showed lower levels of emotional distress. Using path modeling approach, we identified underlying mechanism constructs that explained emotional distress during the COVID-19 pandemic. Our findings indicate a critical need of addressing access to healthcare systems and mental health resources for People of Color, especially younger People of Color, to improve emotional distress.

## Data Availability

The datasets generated and analyzed during the current study are not publicly available to protect identifiable information on the geographic information system (GIS), substance-used related data, and up-hold the consent obtained by participants engaged in this study–approved by the Virginia Commonwealth University Institutional Review Board, however, upon specific request please contact mtadua@vcu.edu for questions about data availability.
